# Stochasticity Highlights the Development of Both the Gastrointestinal and Upper-Respiratory-Tract Microbiomes of Neonatal Dairy Calves in Early Life

**DOI:** 10.3390/ani15030361

**Published:** 2025-01-27

**Authors:** A. Nathan Frazier, Logan Ferree, Aeriel D. Belk, Khalid Al-Lakhen, M. Caitlin Cramer, Jessica L. Metcalf

**Affiliations:** 1 United States Department of Agriculture—Agricultural Research Service (USDA-ARS), Bushland, TX 79012, USA; 2 Department of Animal Science, Colorado State University, Fort Collins, CO 80523, USA; 3 Department of Animal Science, Auburn University, Auburn, AL 36849, USA; 4 Canadian Institute for Advanced Research (CIFAR) Azrieli Global Scholars Program, CIFAR, Toronto, ON M5G 1M1, Canada

**Keywords:** microbial ecology, dairy calf, disease, stochasticity, determinism

## Abstract

The microbiome of dairy calves undergoes extensive change due to various forces during the first weeks of life. Importantly, diseases such as bovine respiratory disease (BRD) and calf diarrhea can have profound impacts on the early-life microbiome. Therefore, a longitudinal, repeated-measures pilot study was designed to characterize the establishment of nasal and fecal microbiomes of dairy calves, assess the governing forces of microbial assembly, and evaluate how disease states impact these microbial ecologies. Dairy calves (*n* = 19) were clinically evaluated for gastrointestinal and respiratory disease across three weeks beginning at age ≤ seven days old. Fecal (*n* = 57) and nasal (*n* = 57) microbial samples were taken for paired-end 16S rRNA gene amplicon sequencing. Taxonomy and diversity analyses were used to characterize early-life nasal and fecal microbiomes. Stochasticity and determinism were measured using normalized stochasticity testing (NST) and Dirichlet multinomial model (DMM). All analyses were tested for statistical significance. Clinical diarrhea was observed in 11 of the 19 calves. Clinical BRD was not independently observed among the cohort; however, two calves presented clinical signs of both BRD and diarrhea. Taxonomic analysis revealed that fecal samples were highlighted by *Bacteroidaceae* (40%; relative abundance), *Ruminococcaceae* (13%), and *Lachnospiraceae* (10%), with changes in diversity (Kruskal–Wallis; *p* < 0.05) and composition (PERMANOVA; *p* < 0.05). Clinical diarrhea reduced diversity in the fecal microbiome but did not impact composition. Nasal samples featured *Moraxellaceae* (49%), *Mycoplasmataceae* (16%), and *Pasteurellaceae* (3%). While no diversity changes were seen in nasal samples, compositional changes were observed (*p* < 0.05). NST metrics (Kruskal–Wallis; *p* > 0.01) and DMM (PERMANOVA; *p* < 0.01) revealed that stochastic, neutral theory-based assembly dynamics govern early-life microbial composition and that distinct microbial populations drive community composition in healthy and diarrheic calves.

## Simple Summary

Many factors influence the development and establishment of the neonatal gut and upper-respiratory-tract microbiomes. Importantly, these microbiomes play a significant role in host maintenance and health physiologies in host–microbe systems. In dairy calf systems, the ecological factors impacting microbial assembly are not fully elucidated. Thus, the aim of this pilot study was to characterize the establishment of early-life fecal and nasal microbiomes in dairy calves and evaluate the impact of disease states on microbial development. In addition, we investigated the governing forces of microbial composition to better understand the factors that shape microbial community populations. Herein, we observed dynamic changes in diversity and composition in the neonatal calf microbiomes and demonstrated that disease state impacts the gastrointestinal microbiome. Our results further suggest that early-life microbiomes are stochastically driven, governed by neutral theory-based dynamics. These results together provide researchers with a roadmap to further investigate how early-life microbiomes develop and how they are affected by disease. Together, this study and future studies could help improve the quality of life in dairy calf management.

## 1. Introduction

Mammals and microorganisms have evolved a fundamental, symbiotic relationship crucial to mammalian life that is known as the microbiome. The microbiome is the collection of all microbes and the genomes that naturally inhabit their host. Host–microbe interactions influence both the host and its microbial partners. Currently, a large effort to characterize animal-associated microbial ecologies has resulted in an improved understanding of the importance of the microbiome [1]. In humans, studies have indicated the importance of the microbiome to health [2,3,4], digestion [5], growth and development [6], and behavior [7,8]. In parallel, recent research has increased the understanding of the microbiomes of domesticated livestock such as dairy cattle. Research efforts in dairy cattle have revealed the relationship between the microbiome and diet [9], methane emissions [10], stress and physiology [11], and overall health [12,13]. The vast importance of the dairy cow microbiome has given way to a significant effort to understand how their microbial partners establish and assemble. In humans, and likely in all mammalian life, microbial establishment is thought to begin at the time of birth via vertical transmission governed by various stochastic forces [14]. Similarly, early-life gut microbiomes in dairy calves assemble in the same stochastic manner with variations in composition due to vaginal birth or Cesarean section birth [15]. While the establishment of the early dairy calf microbiome is beginning to be understood in greater detail [15,16], the colonization of early-life microbiomes is a complex process involving both the host and its surrounding environment. Thus, further investigation is crucial to fully understanding the establishment of the dairy cow microbiome.

Most investigative studies in cattle characterize the microbiome in adults, while the importance of the early-life microbiome has only recently seen an increase in research attention [17,18]. The early-life microbiome could have profound implications for its symbiotic host, as evident in other ruminant livestock functionalities [19]. Many factors influence the establishment of the neonatal microbiome and can be defined as either stochastic or deterministic. Importantly, recent research has indicated that early-life microbial structures could be primarily stochastically driven [15]. In real ecological systems, stochastic forces are often constrained by deterministic processes such as age and diet, leading to a unification of both niche and neutral theory [20,21]. Thus, while neutral theory is inherently stochastic, unified neutral theory can reasonably be applied to real ecological environments. In young ruminants such as dairy calves, there is little evidence supporting the existence of neutrality. Furman et al. [15] demonstrated that the deterministic factors diet and age, and stochastic factors, act in concert to shape the assembly of the rumen microbiome. Additionally, Pan et al. [22] revealed neutral theory-based assembly in neonatal calves with or without clinical diarrhea onset. However, to date, no other studies have directly investigated the stochastic neutrality of microbial assembly, nor have the factors that can affect microbial assembly been fully elucidated.

**Figure 1:**
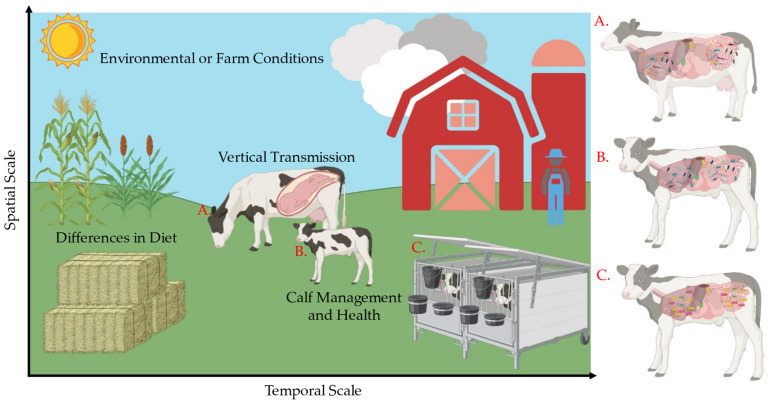
Factors contributing to microbial assembly differences. Stochasticity and determinism are important forces in microbial assembly patterns. Stochastic, random forces such as vertical transmission are important in early-life microbial compositions. Deterministic forces such as diet contribute to composition shifts that often constrain the stochastic forces across a temporal scale. As the calf ages, it will inevitably have a different microbiome (**A**) than it did in early life (**B**). Additionally, factors like calf management, health, and environmental filtering can have marked effects on microbial assembly. Calves reared in hutches (**C**) can have different microbial ecologies than those allowed access to their dam (**B**). These forces are important to the microbial assembly process that is seen in later life.

Many factors can disrupt the establishment of the microbiome, creating altered microbial ecologies (Figure 1). These factors include husbandry, calf management, environmental and/or farm conditions, and diet. Importantly, disease is one of many factors that can perturb microbial assembly and functionality. These altered microbiomes could impact the effort to diagnosis and treat important diseases for the dairy industry. Specifically, pre-weaned dairy calf morbidity and mortality have remained mostly unchanged over the last 18 years due to diseases such as bovine respiratory disease (BRD) and calf diarrhea [23,24,25,26]. Thus, there is a need to develop novel ways to identify animals at a greater risk for disease to address the economic impact of disease on the dairy industry [23]. The microbiome could prove beneficial in addressing these issues by assisting in understanding the role microbes play during perturbation events such as disease. Surprisingly, recent research efforts have reported conflicting findings when assessing the microbiome and disease. In fecal microbiomes, there is evidence of diversity and taxonomic differences between healthy and diarrheic calf microbiomes [27]. In contrast, studies have also reported no changes between healthy and diarrheic calves [28]. Similar patterns can be seen in nasal microbiomes when assessing BRD events in dairy calves [29,30]. Additionally, differences in methodologies between studies creates variation, making comparison between studies difficult. Studies using a defined methodological approach could prove valuable to reducing cross-study variation [31]. Further investigation is warranted and necessary to untangle the cross-study variation in results. Overall, it remains unclear which mechanisms and factors regulate divergent microbial communities during perturbation events such as disease.

While the establishment of the neonatal calf microbiome has received an increase in research interest, there remains a need to investigate the governing processes of microbial community development as well as the impact of disease state in early-life microbiomes. Additionally, many studies are constrained by age in longer-term studies, resulting in diversity changes being attributed to animal age rather than disease state. Thus, investigating temporal patterns on a narrower timescale could elucidate key microbial factors that either are impacted by perturbation events such as disease or have an impact on later development stages. Therefore, we hypothesized that early-life (<3 weeks of life) microbiome establishment is governed by stochasticity and that these forces could be impacted by disease onset. To test this, a pilot study was designed to provide proof-of-concept analysis of the development and assembly of gastrointestinal (GI) and upper-respiratory-tract (URT) microbiomes in dairy calves (the outcome) during the first three weeks of life and to understand the correlations between disease states and their associated microbiomes in early life (the exposure). Our study sought to answer three primary questions: (i.) how microbiome establishment occurs in neonatal calves in early life, (ii.) which forces (i.e., stochastic or deterministic) are driving community assembly during the first three weeks of life, and (iii.) whether disease impacts microbial development during the first three weeks of life. We utilized inexpensive and rapid 16S rRNA gene sequencing to assess GI and URT microbial ecologies and combined this with health assessments to identify calves with respiratory disease and diarrhea.

## 2. Materials and Methods

### 2.1. Animals, Facilities, and Experimental Design

The present study was part of a larger longitudinal, repeated-measures observational cohort recruited from a larger study population investigating respiratory disease at a commercial dairy farm located in Colorado spanning from March to early April 2021 [32]. For this pilot study, the dairy was chosen based on proximity to Colorado State University and previous successful engagement in research. Calf enrollment was not randomized and was based on days of age. Calves were enrolled on a weekly basis as they were born, with the enrollment period beginning 4 March and ending on the 25th of the month. Calves aged ≤ seven days were enrolled in the study at sampling time point one and evaluated for two additional sampling time points. Six calves were enrolled on 4 March, three calves were enrolled on 11 March, six calves were enrolled on 18 March, and seven calves were enrolled on 25 March. Final evaluations occurred on 8 April. Calves were enrolled regardless of health status. This method was used to capture microbiome establishment under various states of health. In total, 19 calves (*n* = 19) were utilized for microbiome analyses. Of the 19 calves used in the study, eight were Jersey, eight were Holstein, and three were cross-bred; additionally, there were ten males and nine females. Calves were immediately removed from the dam following birth and individually housed throughout the pre-weaning period. The calves were not completely isolated, as they were able to see their neighbors. All calves were only fed pooled whole-line milk via a bucket and did not have access to forages during the first three weeks of life. All animal husbandry duties were performed by dairy personnel; however, they were not directly involved in the collection of samples, health assessments, or administration of treatment.

### 2.2. Microbial Sampling and Clinical Health Observations

Microbial sampling was performed by trained research personnel (A.N.F.) at each sampling time point. Each calf was sampled one time per week, which coincided with health exams. The experimental unit was the calf, and all data were collected at the calf level. Sampling consisted of both a nasal and a fecal swab using labelled, double-headed sterile cotton tip swabs (BD 220135; Franklin Lakes, NJ, USA). The nasal swab was inserted into the calf’s left nasal cavity, approximately five to seven cm inside, and nasal contents were gathered from the nasal vault (*n* = 57); fecal swabs were collected by placing a sterile swab approximately five to seven cm into the rectum of the calf and removing fecal content (*n* = 57). Following sampling, the swabs were placed into freezer bags by sample type, immediately placed on ice, transported to a −20 °C freezer located at the Metcalf microbiome laboratory (Colorado State University, Fort Collins, CO, USA), and stored until DNA extraction following all sampling time points. No research personnel involved in sample collection or data analyses were blinded to health status because microbiome data results are not considered subjective.

The calves were observed for clinical health parameters once per week by trained research personnel (L.F.) during the three-week sampling time frame using the Wisconsin Calf Health Scoring System [33]. The Wisconsin Calf Health Scoring System uses the following parameters to assess calf health: nasal discharge, eye discharge, ear position, cough (based on number of coughs per minute, e.g., one cough per minute equated to a score of 1 or mild), rectal temperature, observation of fecal consistency for calf diarrhea, and navel and joint palpation. All parameters were scored from zero to three, with zero being healthy, one being mild, two being moderate, and three being severe. A lung ultrasound was performed, and lung lesions were identified for determination of respiratory disease as previously described [34]. Lung ultrasounds were conducted by one trained research team member to reduce bias and the subjectivity of score reporting (L.F.). Respiratory disease was defined using both clinical observations and lesions found during ultrasound. Calves considered to have respiratory infections were treated with tulathromycin (Draxxin^®^, Zoetis Services, LLC, Parsippany, NJ, USA; 1.1 mg/kg subcutaneously once) during a health exam if one or both of the following were present: (1) two or more abnormal clinical signs and/or (2) the number of lung lesions found was ≥two [32]. Following health scoring at each time point, calves were assigned to one of four health categories: (i). healthy—calves presenting no signs of clinical disease, (ii). BRD—calves presenting clinical signs consistent with respiratory illness, (iii). diarrhea—calves presenting clinical signs consistent with GI disorder, or (iv). BRD plus diarrhea—calves presenting clinical signs of both respiratory and GI disorder. Researchers worked with farm staff to maintain treatment records; however, farm records were unreliable, and therefore, only health information collected by researchers was used for analysis.

### 2.3. DNA Extraction and Sequencing

At the conclusion of the sampling time frame, microbial communities were characterized via paired-end 16S rRNA gene sequencing similar to previously published methods [35]. All DNA extraction was carried out by trained research lab personnel within the Metcalf lab. DNA was extracted from all fecal samples (*n* = 57) and nasal samples (*n* = 57) using the Qiagen PowerSoil Kit (Qiagen 27000-4-KF; Hilden, Germany) per the manufacturer’s instructions, with one modification during the binding step (2 band steps were used to utilize all the supernatant in step 11). DNA was extracted from all fecal and nasal swabs using 96-well plates, with nine negative extraction control samples and three positive mock microbial community controls (ZymoBIOMICS D6300; Irving, CA, USA) per plate. Controls were used to confirm no contamination in the sequencing run (negative controls) and provide verification for sequencing accuracy (positive controls). Once DNA was extracted, the DNA was amplified and sequenced using 515f/806r primers targeting the V4 region of the 16S rRNA subunit following the Earth Microbiome Project protocols (www.earthmicrobiome.org; accessed on 4 March 2021) [36]. Sequences were multiplexed using error-correcting Golay barcodes within the PCR primers and quantified using Picogreen Quant-iT (Invitrogen P7589, Life Technologies; Grand Island, NY, USA). Quantified PCR products were pooled at equimolar concentrations for sequencing. Pools were then sequenced using a 500-cycle kit on the Illumina MiSeq sequencing platform (Illumina; San Diego, CA, USA) on Colorado State University’s campus. Samples were placed in the sequencing wells at random to avoid being confounded due to technical artifacts. The 16S rRNA gene sequencing data are publicly available in the EBI repository under study accession PRJEB76862 and on the QIITA study platform under study ID 13813 [37].

### 2.4. Analysis of Fecal and Nasal Microbiomes

Following sequencing, data processing and analyses were performed using Quantitative Insights into Microbial Ecology (QIIME2) version 2020.8 [38]. Sequence data were demultiplexed, quality filtered, and denoised using the QIIME2 dada2 plugin version 2021.8.0, creating amplicon sequence variants (ASVs) [39,40]. Taxonomic classification was achieved using the SILVA 138 99% database via the QIIME2 feature-classifier plugin, and ASVs assigned to mitochondria and chloroplasts were subsequently filtered [41,42]. Sequences were rarefied to a sampling depth of 9060 ASV/sample to filter low-quality reads based on initial analysis of the sequencing run. Filtering steps were also conducted to remove sequences that appeared in less than 10% of all samples. A phylogenetic insertion tree was constructed via the QIIME2 fragment-insertion plugin, which utilizes the SEPP program, with the SILVA 128 tree used as a reference tree backbone [40,43]. The phylogenetic tree was used in downstream analysis.

Prior to downstream analyses, samples were split into two groups using the QIIME2 filter-samples plugin: fecal and nasal. This was based on the sample type and was carried out to analyze the fecal and nasal microbiomes individually as well as analyze their associations with disease state. Breed and sex were assessed for differences in community composition using unweighted UniFrac distance, with statistical comparisons carried out using permutational multivariant analysis of variance testing (PERMANOVA) with multiple testing correction using the “pairwise” parameter in QIIME2 (q-value), which assesses significant differences between the sample groups and controls false discovery rates (FDR) [44,45]. Linear regression models were fit to establish the correlation between alpha diversity using breed and sex as predictors.

Differential abundance for taxonomic analysis was assessed for both fecal and nasal samples using analysis of composition of microbiomes with bias corrections (ANCOM-BC) across sampling time points [46]. ANCOM-BC analysis measures the differential abundance of taxa between groups while accounting for compositionality and controlling both Type I and Type II error, producing an FDR-adjusted *p*-value (q-value). The top three enriched or depleted differentially abundant bacteria were recorded at alpha level = 0.001. Phylogenetic diversity analysis was run using the core metrics pipeline within QIIME2 [38,42,43]. Alpha diversity was measured for fecal and nasal samples using Shannon’s Index, Faith’s Phylogenetic Diversity (PD), and observed richness metrics [47,48,49]. Briefly, Shannon’s Index measures diversity by accounting for richness (number of species in a sample) and evenness (distribution of species abundances), Faith’s PD considers species richness and their phylogenetic relationships, and observed richness simply counts the total number of unique species. Alpha diversity metrics were then tested statistically using a Kruskal–Wallis test with a Benjamini–Hochberg multiple-testing correction, providing both a *p*-value and an FDR-adjusted *p*-value (fdr_bh) [50]. Linear regression models were fit for both nasal and fecal samples to establish the correlation between alpha diversity, with sampling time point as the predictor, using the “stats” package in R version 4.4.2 and using RStudio version 2023.12.1 [51]. Linear regression models producing significant results were retested using multiple regression models, with sampling time point, calf age, and individual calf as predictors, to increase model applicability. Beta diversity was analyzed for each sample group using unweighted UniFrac distance with PERMANOVA multiple-testing corrections. Fecal samples were further analyzed based on health status. Calves were grouped into either healthy calves or diarrheic calves. Health status was derived from fecal severity scores, where healthy calves corresponded to fecal severity scores of “healthy” or “mild”, and diarrheic calves corresponded to fecal severity scores of “moderate” and “severe”. Taxonomic, diversity, and composition testing was analyzed using the methods described above for health status groups. Linear regression models were also fit for health status using sampling time point as the predictor for any correlations to alpha diversity. All diversity and compositional statistical testing were considered significant at alpha level = 0.05.

Next, normalized stochasticity testing (NST) was performed using the Bray–Curtis distance matrix, providing the modified Raup–Crick distance (β_RC_) [52], the standardized effect size index (SES) [53,54], the normalized stochasticity index (NSTi), and the modified stochasticity ratio (MST) [55]. Since stochastic and neutral metrics rely on abundance-based comparisons, Bray–Curtis was chosen, as it captures abundance changes better than unweighted UniFrac distance. These metrics were calculated in R using cNST within the “NST” package to determine the stochastic or deterministic nature of assembly in both fecal and nasal microbiomes following similar previously published methods [22,55]. For β_RC_, values approaching zero indicate stochastic, random assembly driven by neutrality. The magnitude of the SES index determines the strength of deterministic forces on community assembly such that SES values > 2 and <−2, indicating deterministic processes, are favored. Like β_RC_, values close to zero indicate neutrality. For NSTi, values greater than 0.5 indicate stochasticity and values approaching 1 indicate that stochastic processes are the primary drivers of community assembly. Similarly, MST provides a percentage where >50% favors stochasticity. Nonmetric multidimensional (NMDS) scaling plots were used to visualize the ordination of community compositions by β_RC_ values. NSTi, MST, SES, and β_RC_ fecal values were tested for significance across sampling time points and health status using Kruskal–Wallis with Benjamini–Hochberg multiple-testing corrections [50]. Failing to reject the null model (*p* > 0.01) indicates that the values are not changing across time. A significance of alpha level 0.01 was used to reduce the risk of false-positive rates, as microbiome data can contain high variability. This adjustment ensured that only robust and biologically meaningful differences were considered significant. Only the sampling time point was used for nasal samples.

We then evaluated whether different taxa were driving microbial community structure for healthy calf and diarrheic calf groups. Fecal samples were filtered into either the healthy or the diarrheic group using the QIIME2 filter-samples plugin and analyzed. The Dirichlet multinomial model (DMM) was used in R (“DirichletMultinomial” package) to identify key changes in microbiome development at the genus level. The DMM method utilized the lowest Laplace approximation score to cluster samples based on microbial structure similarity. The top three genera with the highest approximation scores were considered main drivers of microbial community structure for each group (i.e., healthy and diarrheic) [22,56,57]. The clusters were tested for significance against the Bray–Curtis distance matrix with PERMANOVA using the adonis2 function within the “vegan” package in R [58,59]. PERMANOVA adonis2 testing used 999 permutations to mitigate Type I error by generating a null distribution. Significant results (*p* < 0.01) demonstrate that the clusters derived from the DMM are not random and reflect true differences in community structure. Thus, significant results indicate that the top three genera indeed have a role as primary drivers. Similar to NST testing, alpha level was set at 0.01 to reduce false-positive rates, ensuring only biologically meaningful differences were captured. All visualizations were performed with R using the “ggplot2” package in RStudio [60].

## 3. Results

### 3.1. Calf Characteristics and Sequencing Results

Nineteen calves were swabbed for fecal and nasal microbiome characterization as well as observed for clinical health assessment (Table 1) over a three-week sampling time frame. A total of 22 calves were initially screened and sampled for analysis; however, three calves were removed from the study due to lameness, farm management errors by farm staff, and repeated antibiotic treatment. For the analyzed population (*n* = 19), calf breed, sex, and health characteristics were recorded. For breed, there were eight Jersey, eight Holstein, and three cross-bred calves enrolled in this study; additionally, there were ten males, and nine females enrolled. Overall, there were 17 incidences of observed clinical disease. A total of 11 calves were observed with clinical diarrhea, with four calves having clinical signs at two or more sampling time points. At time point one, clinical signs of GI illness (i.e., diarrhea) were observed for three calves only. All other calves were reported healthy. At time point two, a distinct increase could be noticed for observed clinical disease. Clinical signs of diarrhea were observed in eight calves while 10 remained healthy. BRD/diarrhea was reported in one calf. At time point three, clinical signs of diarrhea were observed in four calves, while once more, one calf was reported to show clinical signs of BRD/diarrhea. The remaining 14 calves were reported to be healthy.

In total, there were 114 combined samples taken for microbial analysis. Paired-end sequencing yielded 7,063,380 sequencing reads. Denoising, quality filtering, removing chimeras, and filtering steps yielded a total of 3425 unique ASVs, with a total frequency of 4,016,483 with a mean feature frequency per sample of 35,232.30 (range: of 209 to 59,874 ASVs/sample). Quality-control checks of negative controls indicated low sequencing numbers (0–285 feature count), signaling contamination of samples during DNA extraction was not likely. Based on the positive control taxonomic mock community, the sequencing run was determined to be valid (Figure S1). Quality-control checks of the positive and negative sample controls indicated it was reasonable to remove control samples and utilize the data to answer the three proposed questions.

### 3.2. Characterization of the Early-Life Microbiome

**Figure 2:**
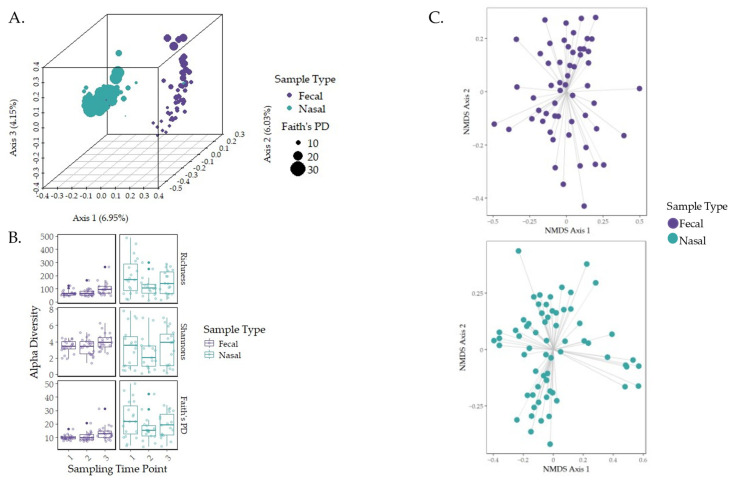
Alpha and beta diversity metrics for nasal and fecal samples over the sampling time frame. Community assembly was also assessed. (**A**) Beta diversity of each sample type distance was measured using unweighted UniFrac distance and revealed that separation was driven between sample type. (**B**) Alpha diversity was measured using Faith’s PD, Shannon’s Index, and richness, with statistical significance analyzed using Kruskal–Wallis pairwise testing across the three sampling time points [61]. (**C**) The ordination of community compositions based on β_RC_ dissimilarity distance is represented by NMDS plots.

Fecal and nasal samples had distinct microbial compositions (Figure 2A; unweighted UniFrac PERMANOVA, *p* = 0.001, q = 0.001), with nasal samples having higher diversities (*p*-value < 0.05). Therefore, for further analysis, the fecal and nasal samples were separated. Analysis of sex and breed using PERMANOVA revealed a significant difference for breed in fecal samples between cross-bred calves and Holstein calves (*p* = 0.045, q-value = 0.0675 corrected for multiple comparisons) and between cross-bred calves and Jersey calves (*p* = 0.019, q-value = 0.0570). For fecal data only, PERMANOVA analysis revealed no significant differences in microbial communities for sex. For nasal data only, PERMANOVA analysis revealed no significant difference in microbial communities for sex; however, a significant difference was observed for breed between cross-bred calves and Holstein calves (*p* = 0.004 q-value = 0.006 corrected for multiple comparisons) and cross-bred calves and Jersey calves (*p* = 0.003, q-value = 0.006). Unfortunately, the small sample size of cross-bred calves prevented the consideration of breed as a factor within this study. Additionally, linear regression models indicated that breed (Figures S2A and S3) and sex as predictors (Figure S4) were not correlated with diversity metrics or changes.

**Figure 3:**
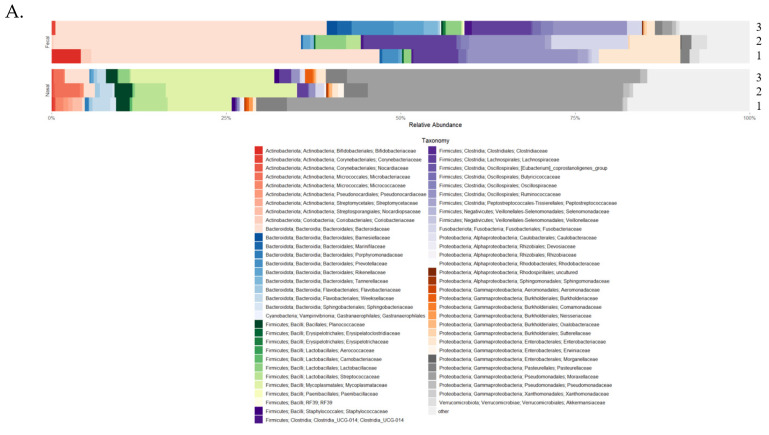

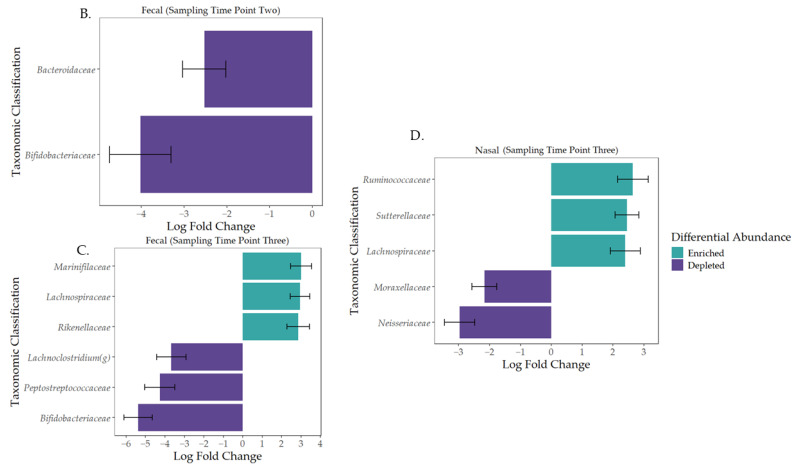
Taxonomy and taxonomic analysis using differential abundance testing (ANCOM-BC). (**A**) Taxonomic profiles for both fecal and nasal microbiomes using mean relative abundance at the family level across the three sampling time points (1–3) [61]. (**B**) Differentially abundant fecal taxa at sampling time point two. (**C**) Differentially abundant fecal taxa at sampling time point three. (**D**) Differentially abundant nasal taxa at sampling time point three.

The taxonomic profiles, diversity levels, and compositions for fecal and nasal samples were analyzed over the first three weeks of life. *Bacteroidaceae* (40%), *Ruminococcaceae* (13%), *Lachnospiraceae* (10%), *Enterobacteriaceae* (7%), and *Fusobacteriaceae* (5%) were the most relatively abundant bacteria in fecal samples at the family level throughout the sampling time frame (Figure 3A). Additionally, *Bifidobacteriaceae* (6%) was higher in relative abundance at time point one. ANCOM-BC analysis at the ASV level revealed that *Bacteroidaceae* and *Bifidobacteriaceae* were depleted at sampling time point two (Figure 3B; q-value < 0.001). At sampling time point three, *Marinifilaceae*, *Lachnospiraceae*, and *Rikenellaceae* were the top three enriched families, and *Bifidobacteriaceae*, *Peptostreptococcaceae*, and *Lachnoclostridium* (*genus*) were the top three depleted bacterial groups (Figure 3C; q-value < 0.001). Fecal diversity changes were detected during the first three weeks of life (Figure 2B). The fecal sample microbiota was different in terms of Faith’s PD (*p*-value = 0.001, fdr_bh = 0.004) and richness (*p*-value = 0.003, fdr_bh = 0.008) between sampling time points one and three. Between sampling time points two and three, Faith’s PD (*p* = 0.003; fdr_bh *p* = 0.005) and richness (*p* = 0.007; fdr_bh *p* = 0.001) were significantly different, indicating a change in alpha diversity. Correlation analysis using linear regression models using sampling time point as a predictor indicated that sampling time point two was significantly correlated with fecal diversity changes in Faith’s PD only (Figure S5A; *p*-value = 0.002, R^2^ = 0.20). Given the low R^2^ value of the previous model, a second multiple regression model was generated, with the predictors being sampling time point, individual calf, and age in days, to determine the influence of multiple predictors on Faith’s PD. The new model indicated that these predictors were not correlated with Faith’s PD (Figure S6A; *p*-value = 0.3033, R^2^ = 0.57).

The most relatively abundant bacteria in nasal samples were the families *Moraxellaceae* (49.42%), *Mycoplasmataceae* (16.24%), *Pasteurellaceae* (3.4%), *Streptococcaceae* (2.83%), and *Weeksellaceae* (2.06%; Figure 3A). By week three, *Mycoplasmataceae* and *Bacteroidaceae* had increased in relative abundance (24.85% and 4.1%, respectively). ANCOM-BC analysis at the ASV level revealed that *Ruminocccaceae*, *Sutterellaceae*, and *Lachnospiraceae* were the top three enriched families, while *Neisseriaceae* and *Moraxellaceae* were depleted at sampling time point three (Figure 3D; q-value < 0.001). Alpha diversity and composition analysis produced non-significant results. However, linear regression analysis indicated that sampling time point as a predictor was correlated with nasal diversity changes in Faith’s PD (Figure S5B; *p*-value = 0.0003, R^2^ = 0.22) and richness (*p*-value = 0.0005, R^2^ = 0.2) at sampling time point two. As with fecal samples, new multiple regression models were generated due to the low R^2^ values of the previous models, with the predictors being sampling time point, individual calf, and age in days. The new regression models revealed that the predictors were not correlated with Faith’s PD (Figure S6B; *p*-value = 0.2369, R^2^ = 0.59); however, the predictors were correlated with richness (Figure S6B; *p*-value = 0.016, R^2^ = 0.72).

### 3.3. Governing Forces of Microbial Community Composition

There was a difference in microbial composition in fecal samples between sampling time points one and two (unweighted UniFrac PERMANOVA, *p* = 0.044, q = 0.044), one and three (*p* = 0.001, q = 0.001), and two and three (*p* = 0.001, q = 0.001). The changes in community composition were analyzed for the forces driving community assembly. Fecal NSTi, MST, SES, and β_RC_ revealed values of 0.9765, 54.91%, 0.0576, and 0.0146, respectively (Table S1). The high NSTi value indicates that stochasticity dominated community assembly, while the MST value of 54.91% indicates that community assembly favored stochastic forces. The SES and β_RC_ values confirm that community assembly favored neutrality (Figure 2C). Kruskal–Wallis testing revealed non-significant results for each of the values when tested against sampling time points (*p* > 0.01; Table 2).

Within nasal samples, microbial community composition differed between sampling time points one and two (unweighted UniFrac PERMANOVA, *p* = 0.038, q = 0.057) and time points one and three (*p* = 0.004, q = 0.012). The nasal NSTi, MST, SES, and β_RC_ values were 0.9603, 36.61%, 0.0463, and 0.0166, respectively (Table S1). The high NSTi value indicates dominance of stochastic forces driving community assembly. While the MST value of 36.61% indicates that deterministic factors were favored in nasal community assembly, the model emphasizes the observed versus the null model such that it can downplay stochasticity in datasets where deterministic processes are favored in some sample pairs. Furthermore, the SES and β_RC_ values likely confirm neutral processes in both fecal and nasal community assembly (Figure 2C). Kruskal–Wallis testing in nasal samples also returned non-significant results (*p* > 0.01) for the NST testing values when tested against sampling time points (Table 2).

### 3.4. Impacts of Disease on Early-Life Microbial Establishment

**Figure 4:**
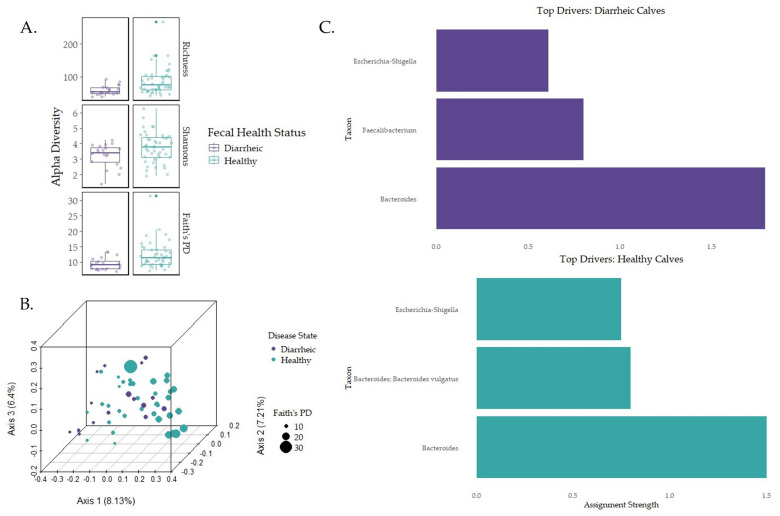
Alpha and beta diversity metrics for healthy and diarrheic calves with the top three community drivers at the genus level. (**A**) Alpha diversity metrics were measured for fecal samples and health status. Statistical analysis was conducted with Kruskal–Wallis pairwise testing. (**B**) Unweighted UniFrac distance was used to analyze beta diversity for fecal samples and health status and tested for significance using PERMANOVA pairwise analysis [61]. (**C**) DMM generated the top three genera for microbial community assembly using the lowest Laplace approximation score for microbial structure similarity clustering.

Early-life microbiome association with disease state was only assessed for diarrhea and fecal microbiomes given the low incidence rate of BRD-positive calves. Table S2 details the number of calves associated with fecal severity scores used in the analysis. Calves with “healthy” or “mild” fecal scores were considered healthy, while calves with “moderate” or “severe” fecal scores were considered diarrheic. In healthy calves, *Bacteroidaceae* (39.52%), *Ruminococcaceae* (13.06%), *Lachnospiraceae* (10.14%), *Enterobacteriaceae* (7.16%), and *Fusobacteriaceae* (3.53%) were the most relatively abundant bacteria at the family level. In diarrheic calves, *Bacteroidaceae* (41.42%), *Ruminococcaceae* (11.94%), *Enterobacteriaceae* (9.35%), *Lachnospiraceae* (9.12%), and *Lactobacillaceae* (5.77%) were the most relatively abundant families. Healthy calves reported a higher relative abundance in *Prevotellaceae* (3.52%) compared to diarrheic calves. Richness was higher in healthy calves compared to diarrheic calves (*p* = 0.002, q = 0.002), phylogenetic diversity (Faith’s PD) was higher for healthy calves compared to diarrheic calves (*p* = 0.0044, q = 0.0044), and Shannon diversity was higher in healthy calves compared to diarrheic calves (*p* = 0.043, q = 0.043; Figure 4A). Multiple regression analysis indicated that fecal severity was not correlated with diversity changes (Figure S7). No compositional differences (PERMANOVA) or enriched features (ANCOM-BC) were detected (Figure 4B; *p* > 0.05). The DMM indicated that the genera *Bacteroides* (two features, with one feature corresponding to the species *Bacteroides vulgatus*) and *Escherichia-Shigella* in healthy calves (Figure 4C). In diarrheic calves, the top drivers were the genera *Bacteroides*, *Faecalibacterium*, and *Escherichia-Shigella*. PERMANOVA testing using the adonis2 function revealed that the relationship between DMM clusters and composition were statistically significant (F-statistic = 8.7342; *p* = 0.001). NSTi, MST, SES, and β_RC_ values tested for significance using Kruskal–Wallis with Benjamini–Hockberg multiple-testing corrections revealed non-significant results (Table 2; *p* > 0.01), indicating the top three genera in each group were possible assembly drivers.

## 4. Discussion

The early-life microbiome is an important component to host maintenance and overall fitness in dairy calves. The assemblage of the dairy calf microbiome plays an important role in the outcome of host physiologies and functionalities [9,10,11,12,13]. Stochastic and deterministic forces can have profound effects on microbial composition, although these forces are not fully understood. Stochastic forces are those that randomly shape community structure and can be probabilistic with respect to species identity or functionality (i.e., historical contingency, drift, dispersal); on the other hand, deterministic forces are those that shape microbial assembly in a defined, predictable manner (i.e., age, diet, host genetics) [19,20,21]. The various factors governed by these forces can impact microbiome assembly and, ultimately, function (Figure 1). In the current study, early-life microbial ecologies varied in both fecal and nasal samples. Importantly, our findings suggest that stochastic processes, as described by neutral theory-based dynamics, may play a significant role in shaping microbial structure regardless of health status within the first three weeks of life. Neutral theory posits that all species have ecological equivalence and that community assembly is driven by random processes. Moreover, previous research supports our results indicating stochastic assembly during the first three weeks of life [22]. Our results indicate that the assembly of the microbiomes is predicated by important microbial community drivers. These microbes shape community populations in fecal and nasal microbiomes, and could be governed by stochastic, neutral theory-based dynamics.

Similar to previous work, the current study revealed that *Bacteroidaceae*, *Ruminococcaceae*, *Lachnospiraceae*, *Enterobacteriaceae*, and *Fusobacteriaceae* were the most relatively abundant families in the early-life GI microbiome regardless of health status [16,28,62]. These findings are indicative of the early, pioneering colonizers within the ruminant GI tract [16]. *Bacteroidaceae* was the most dominant family throughout the first three weeks of life, confirming past results [15,16]. It has also been reported that *Bacteroidaceae* dominate fecal microbiomes during the first two weeks of early life and decrease in abundance as the calf ages [16,28]. Our results corroborate these past findings, as *Bacteroidaceae* was depleted at sampling time point two. Importantly, our results indicate that regardless of health status, features identified in the genus *Bacteroides* were found to be top drivers for community structure assembly (DMM; *p*-value < 0.01). Supporting our findings, the genus *Bacteroidetes* has been well studied in its ability to maintain gut health and has shown the potential to be a biomarker in healthy cattle GI tracts [28]. Taken together, a high relative abundance of *Bacteroidaceae* indicates its importance to both GI health and shaping community structure in the calf early-life microbiome.

Additionally, the families *Ruminococcaceae* and *Lachnospiraceae* have the ability to produce butyrate and have been linked to gut health [62]. Butyrate is a short-chain fatty acid and is a primary source of energy for the rumen epithelium, which is necessary for nutrient and water absorption; butyrate can have beneficial side effects when supplemented in the diet of early-age livestock animals [12]. While not among the top three drivers of community structure, recent evidence has indicated that *Ruminococcaceae* can be predictive of microbial community assembly in both healthy and diarrheic calves [22]. Interestingly, recent findings reported an increase in the fiber-degrading family *Lachnospiraceae* as the calf aged [15]. Similarly, our results indicated that *Lachnospiraceae* was enriched at sampling time point three. Interestingly, our study also reported that *Rikenellaceae* was enriched at sampling time point three. *Rikenellaceae* is thought to be a beneficial bacterial group and has been shown to potentially improve host health and metabolism [63]. Thus, important shifts in microbial ecology are present in neonatal microbiomes, perhaps to prepare the calf for more complex diets. Like Furman et al. [15], no dietary changes were introduced to the calves, suggesting that other factors, such as niche modification by early-arriving species, were involved in microbial assembly. Interestingly, previous research has provided evidence for niche modification, as early-arriving aerobes and facultative anaerobes consume the oxygen, providing an environment for anaerobic species to thrive in the rumen [64]. Overall, our findings could indicate a shift in the microbiome to prepare the animal for solid, fiber-based diets prior to its introduction.

The early-life dairy calf microbiome is characterized by increasing diversity with age and change in diet, especially in the gut microbiome. Similar to previous findings, the current study revealed significant changes to diversity, as measured by Faith’s PD and richness (*p*-value < 0.05) [65,66]. The change in diversity over time validates the dynamic shift in microbiota during the early life of dairy calves. Similarly, the current study revealed that *Bifidobacteriaceae* decreased in relative abundance in fecal samples over time (ANCOM-BC). Given the restrictive nature of on-farm feeding protocols to promote weaning, our results support previous findings in explaining the decrease in *Bifidobacterium* as the calf matures [67]. Bifidobacterium has a well-established role in mammalian gut health, as it provides mechanisms in gut mucosal barrier maintenance by production of beneficial metabolic substrates as well as immunological properties that prevent the attachment of pathogenic organisms [68,69]. The decrease in *Bifidobacterium* over time further highlights the shift to a microbial structure prepared for fiber-based diets. These observed shifts in microbial diversity raise critical questions about the underlying processes driving these changes, particularly the balance between stochastic and deterministic forces. The observed shifts in diversity and composition highlight the dynamic nature of microbial assembly during the first three weeks of life. To better understand the drivers of these changes, we evaluated the role of stochastic and deterministic processes in shaping microbial communities.

Importantly, our results suggest that stochastic forces dominate early-life microbial assembly in the GI microbiome, confirming previous findings [15,22]. The high NSTi and MST values (0.9765 and 54.91%, respectively) suggest that stochastic drivers shape the assembly and structure of early-life microbial populations, providing plausible explanation to observed diversity shifts over time. As an example, Kim et al. [28] reported that *Enterobacteriaceae* was significantly abundant in vaginally birthed calves aged one to four weeks, suggesting vertical transmission (i.e., stochasticity). Similarly, the elevated abundance of *Enterobacteriaceae* reported in the current study could be due to stochastic forces such as vertical transmission, given its place among the pioneer GI colonizers. Interestingly, the near-neutral values of SES and β_RC_ (0.0576 and 0.0146, respectively) indicate that the observed community assembly patterns align with predictions of neutral theory [20]. These findings suggest that the neutral theory of microbial assembly could be governing microbial assembly in the early microbiome. Only two studies have previously reported neutral-based assembly dynamics in the rumen microbiome [15,22]. Therefore, the current study could be among the first studies in suggesting the neutral-based dynamics of microbial assembly in early-life dairy calves. The consistency of diet in the current study validates earlier work of age-dependent assembly mechanisms in early-life microbial structure [15]. Further, microbial composition displayed significant differences between all sampling time points (PERMANOVA; *p*-value < 0.05), supporting the dynamic nature of early-life microbial assembly. Contrary to previous studies that have been confounded by diet when analyzing diversity shifts in dairy calves [65], the calves in our study were only fed line milk. Thus, the diversity and compositional changes within our study were not due to dietary alterations. While our results indicated no correlation between age and sampling time point with alpha diversity increases (*p*-value > 0.05, R^2^ = 0.57), the shifts in composition suggest that age and development of the dairy calf (i.e., determinism) could be major drivers of compositional changes regardless of the influence of diet. This inference is supported by Ma et al. [62], wherein calves fed a regulated diet of milk replacer had distinct compositional shifts during the first eight weeks of life. These findings suggest that as calves age, deterministic forces increasingly shape community dynamics, signifying a gradual shift from stochastic to more deterministic assembly processes. Past studies support this claim, as a transition to more deterministic assembly dynamics was seen between weeks two and three in neonatal calves [22]. However, non-significant results from Kruskal–Wallis testing for NST, MST, SES, and β_RC_ across time points indicate that stochastic and neutral dynamics dominate consistently without temporal variability (*p* > 0.01). Therefore, while the calf might be shifting to more deterministically driven community assembly patterns due to calf age, stochastic forces remain the top drivers of population structure in the first three weeks of life. Our results strongly align with the hypothesis that neutral-based, stochastic forces govern early-life microbial assembly in the GI tract. While our results indicate that stochasticity dominates early microbial assembly and establishment, the current study does not analyze the impact of microbial genetic mutation on community assembly. Therefore, further investigation is needed to validate these claims of neutral-based stochasticity in the first three weeks of life.

While healthy calves demonstrated stochastic assembly dynamics, disease states introduced additional complexity. Diarrheic calves displayed distinct microbial patterns, suggesting that perturbations may shift the balance toward deterministic assembly. Diarrheic calves differed in the most relatively abundant taxa due to the addition of *Lactobacillacae* (5.577%). *Lactobacillus* species are dominant in neonatal calf GI microbiomes, as it is among the microbiota to utilize milk nutrients; thus, the higher relative abundance in diarrheic calves might not correlate to disease state [28]. However, alpha diversity was significantly higher in healthy calves compared to diarrheic calves (*p*-value < 0.05), confirming previous research [70]. Our study highlights that lower diversities are indicative of microbial changes due to perturbations, translating to Anna Karenina principles (AKP), which suggests that dysbiosis is due to stochastic effects rather than deterministic [19,71]. AKP states that “all healthy microbiomes are similar; each dysbiotic microbiome is dysbiotic in its own way.” Our results support AKP, as the DMM demonstrated distinct clustering between healthy and diarrheic calves, indicating that community assembly was driven by distinct microbiota. However, the stochastic principles that create the dysbiotic community structure transition to more deterministic forces once clinical disease is observed. Previous work indicates that deterministic forces are predominant in lower-diversity microbial populations [72]. As the diversity was lower in diarrheic calves in the current study, stochastic forces may have been downplayed in diseased animals. This claim is demonstrated within the current study, as the genus *Faecallibacterium* was among the top drivers of community assembly in diarrheic calves, supporting previous studies indicating that *Faecallibacterium* was highly influential in unhealthy calves at two weeks of age [22]. This family of microbes is also among the butyrate-producing bacteria in the neonatal gut and can induce T-regulatory cells critical for host gut immunological homeostasis [22,73]. Interestingly, *Escherichia-Shigella*, an etiological agent for diarrhea, was among the top drivers in both healthy and diarrheic calves. Because pathogenic bacteria can interact with beneficial community members (e.g., *Faecallibacterium*), resulting in dysbiosis, bacteria such as *Escherichia-Shigella* are thought to be keystone taxa [22]. Thus, our results could potentially support previous findings that unhealthy animals favor deterministically driven assembly, contrasted with healthy calves, which favor stochastically driven assembly. While disease could significantly impact community assembly dynamics, the exact interaction mechanisms between *Faecallibacterium* and *Escherichia-Shigella* were not investigated in the current study. However, PERMANOVA adonis testing indicated that the top three genera in each group were likely valid community drivers (*p* < 0.01). Thus, the difference in community composition between healthy and diarrheic calves are driven by distinct microbial compositions, as supported by previous research [22]. The exact mechanisms for this distinction were not directly tested within this current study. Therefore, these results highlight how disease can shift early-life microbiomes to deterministically driven assembly patterns rather than stochasticity in the GI tract. As posited by Vellend et al. [20], niche and neutral theories can be considered together, and therefore encompass a unified neutral theory capable of explaining both the stochastic and the deterministic nature of microbial assembly. These findings emphasize a potential unified neutral theory and highlight the need for further research in the mechanisms underlying these shifts.

While the fecal microbiome provides insights into GI-related microbial dynamics, the nasal microbiome offers a complementary perspective on host-associated microbial assembly, particularly in relation to respiratory health and microbial transmission in neonatal calves. The nasal microbiome is a key component of the URT and could provide additional insights into early-life microbial assembly processes and their implications for health and disease dynamics. Similar to previous findings, the most relatively abundant bacteria in nasal samples were the families *Moraxellaceae*, *Mycoplasmataceae*, *Pasteurellaceae*, *Streptococcaceae*, and *Weeksellaceae* [30,74,75,76]. Our study was unable to directly test nasal samples with disease state due to the low incidence of BRD-positive animals; however, certain aspects of our findings can be speculated upon with the current literature. As with GI microbial communities, pathogenic and beneficial bacteria inhabit the nasal microbiome [27]. The route of transmission of *Moraxellaceae* and *Streptococcaceae*, commonly associated with pneumonia and otitis media in calves, might contribute to vertical transmission. Our results demonstrate that the nasal microbiome favors stochastic assembly with no temporal variation (NSTi value = 0.9603, *p* > 0.05), as potentially seen in previous studies [30,74,75,76]. It was demonstrated that 63% of microbial composition was shared between the neonatal calf nasal microbiome and the mother’s vaginal microbiome, supporting our claim of stochastic assembly in early-life microbial assembly [74]. Moreover, similar to our findings, *Mycoplasmataceae* has been linked to both sick and healthy animals [30]. While health status could not be directly tested in the current study, as mentioned above, our findings could suggest that pathogens such as *M. dispar* interact with beneficial bacteria in a stochastic manner, creating dysbiosis sufficient for causing disease states (i.e., AKP) [30]. Interestingly, the current study identified that the families *Neisseriaceae* and *Moraxellaceae* were depleted within the nasal microbiomes. Given that the importance of *Neisseriaceae* in URT microbiomes has yet to be determined, its introduction from oral microbiomes suggests stochasticity [77]. More importantly, the depletion of *Moraxellaceae* at sampling time point three suggests that neonatal calf health was improving. Given our study saw little to no clinical evidence of BRD, these results suggest host–microbe interactions in early-life URT microbiomes supporting animal health. These dynamics indicate a shift from stochasticity to more host-derived determinism with immunological consequences.

Furthermore, no diversity changes were detected for nasal samples across time (*p*-value > 0.05), consistent with previous findings [30,76]. The lack of diversity change coupled with composition changes over time (PERMANOVA *p*- value < 0.05) supports our findings that neutral processes are present and do not vary temporally (SES = 0.0463, β_RC_ = 0.0166, *p* > 0.05 respectively). There is little evidence to support these claims within the literature, making our study among the first to report potential unified neutral-based dynamics in the nasal microbiome. Supporting this hypothesis, deterministic properties are present, as MST testing reported that only 36.61% of microbial assembly was explained by stochastic forces, with no temporal variation. Diversity can remain unchanged if stochastic events such as disease are minimal, leading to community structures defined by deterministic forces [19]. Furthermore, environmental filtering can impact microbial assembly. While the calves were individually housed, limiting animal-to-animal contact, the calves could interact with their neighbors as well as the environment. Linear regression models demonstrated that richness was correlated with time in the current study (*p*-value = 0.016, R^2^ = 0.72). An increase in unique microbial members could imply that environmental filtering may have contributed to the observed compositional changes in the nasal microbiome. Environmental filtering can occur through many routes, including differences in husbandry, hygiene, and animal contact. Previous research found that nasal samples from dairy calves were dominated by *Mycoplasma* spp. due to a high prevalence of *Mycoplasma* spp. found on the farm within the study, supporting environmental filtering within the current study [78]. Moreover, *Lachnospiraceae* was enriched at sampling time point three in both fecal and nasal microbiomes. The corresponding enrichment of *Lachnospiraceae* between fecal and nasal microbiomes further supports environmental filtering of URT microbial populations. Overall, the high NSTi value (0.9603), the near-neutral SES and β_RC_ values, and the lack of associated temporal variation provide ample evidence for stochastic-based assembly consistent with unified neutral processes occurring. Both stochastic (e.g., vertical transmission) and deterministic (e.g., environmental filtering) processes may influence early microbial assembly in the URT. Therefore, further evaluation must be undertaken to untangle the balance between stochastic and deterministic processes in URT microbiome assembly.

While there are several strengths of the current study, it is not without its faults. First, multiple breeds were included. Future studies should include enough calves to control for breed statistically since commercial dairies may have more than one breed. Additionally, the number of calves enrolled in the study along with the limited number of sampling time points should be increased in future studies. Increasing the sample population and adding more sampling time points could be better suited for capturing the dynamics of microbial assembly. An increase in sample size and sampling time could also elevate the potential of disease occurrence, primarily in clinical BRD. Larger sampling populations would offer a more robust evaluation of disease state on microbial establishment and assembly.

Second, the farm’s treatment protocol did not include the use of antimicrobials for treating diarrhea, although antibiotics were used for treating respiratory disease. This disparity in antimicrobial use was not assessed in the current pilot study and should be considered in future studies. Past research indicates that antimicrobials can affect microbial ecology in early life. Ma et al. [62] demonstrated that the use of antimicrobials delayed the temporal development of microbial diversity, suggesting that therapeutic use of antimicrobials negatively affects microbial development more so than diarrhea incidence in the first 21 days of life for dairy calves. While calves with two or more antibiotic treatments were removed from the current study, it is unclear which consequences arose from initial antibiotic treatment. Additionally, while past studies have reported no differences in community composition and diversity due to antibiotic treatment, others have reported significant changes in treated calves [76,79]. Thus, future studies should account for antibiotic treatment effects on microbial assembly in both GI and URT microbiomes.

Third, empirical studies face challenges when assessing stochastic effects on microbial community assembly. Primarily, mathematical calculations of within-group distance can include variance due to error and environmental factors that are not measured, resulting in an overestimation of stochasticity [21]. Furthermore, measuring microbial dispersal is often not possible in real-time datasets, making it difficult to measure the influence of generalists across studies [21]. Theoretical models such as the Decomposition Model of Enzymatic Traits (DEMENT) can be employed to better understand the complexity of microbial assembly [80]. Additionally, designing empirical studies with a more complex theoretical framework could provide more robust approaches to untangling the complexity of microbial assembly [19]. Therefore, future studies on the balance of stochastic and deterministic forces in microbial assembly should aim to incorporate many of these elements to better substantiate the hypotheses currently expressed in the pilot study.

Fourth, future studies should investigate the functional aspects of early-life dairy calf microbiomes and dysbiosis caused by disease. Multi-omics microbiome data sets that include combinations of metagenomic and metatranscriptomic sequencing, proteomics, lipidomics, and metabolomics, as well as immune measures, can provide valuable insights into the relationship between microbiomes and disease states. Past research has utilized functional analysis to study various microbial communities in dairy cattle. For instance, Pitta et al. [81] provided metagenomic evidence that metabolic functions within the rumen microbiome varied in three different treatment groups of dairy cows in response to age and physiological changes. Incorporating functional analyses could enhance our understanding of how stochasticity and deterministically driven assemblies occur in nature. These methodologies could untangle the functional potential with observed taxonomic patterns. Additionally, functional analyses could help tease apart the dynamic nature of the role of disease state in microbial assembly and composition. Future studies should aim to include functional analysis to quantify the mechanisms of microbial assembly during perturbation events. Despite these limitations, our study adds valuable insights into early-life microbial assembly and highlights critical areas for future investigation.

## 5. Conclusions

Early-life microbiomes in calves are important for the development of the animal and maintenance of health. The current pilot study characterizes the establishment of fecal and nasal microbiomes during the first three weeks of life of dairy calves as well as investigates the forces that drive community assembly. Our study is among the first to potentially describe stochastic, unified neutral theory-based assembly dynamics in both GI and URT microbiomes. Stochastic processes seem to dominate in both GI and URT microbial assemblies; however, evidence suggests that deterministic factors may reduce stochasticity in nasal microbiomes. Additionally, distinct microbial compositions are seen in between healthy and diarrheic calves and are driven by different microbial members. However, given the limited sample size and sampling time points, larger studies are necessary to support this hypothesis. Functional analysis could further validate the dynamics involved in early-life microbial assembly. Overall, this study provides a foundational framework for understanding microbial assembly in neonatal calves, offering novel insights into the interplay between stochastic and deterministic processes. In addition to understanding assembly dynamics, our findings on clinical diarrhea emphasize the importance of investigating perturbations and their implications on microbial ecology. Our findings suggest that healthy and diarrheic calves have distinct microbial clusters that drive community composition, indicating that perturbation could be evident of AKP. Our findings provide a roadmap for future research into neonatal calf microbial ecology, with critical implications for improving calf health and understanding microbial dynamics across host-associated environments.

## Figures and Tables

**Table 1:** Calf breed and sex characteristics group by health category ^1^.

Health Category *	Time Point 1	Time Point 2	Time Point 3
**Healthy**	16	10	14
*Breed*			
*Jersey*	*7*	*3*	*7*
*Holstein*	*6*	*5*	*5*
*Cross*	*3*	*2*	*2*
*Sex*			
*Male*	*9*	*4*	*8*
*Female*	*7*	*6*	*6*
**Diarrhea**	3	8	4
*Breed*			
*Jersey*	*2*	*5*	*2*
*Holstein*	*1*	*2*	*2*
*Cross*	*0*	*1*	*0*
*Sex*			
*Male*	*2*	*6*	*2*
*Female*	*1*	*2*	*2*
**BRD** ^¥^	0	0	0
**BRD/Diarrhea**	0	1 ^¶^	1 ^!^

^1^ [61], * Health categories were determined by scores from the Wisconsin Calf Scoring System [33]. ^¥^ No observations of clinical BRD were recorded. ^¶^ The only calf observed with both clinical diseases was a Jersey male. ^!^ The only calf observed with both clinical diseases was a cross-bred male.

**Table 2:** Significance results of Kruskal–Wallis with Benjamini–Hockberg multiple testing corrections (*p*-value, with adjusted p-value in parentheses) from cNST testing from both sampling time point and disease state.

Sample Type		NSTi	MST	SES	β_RC_
Fecal	Sampling time point	0.3382 * (0.5411)	0.3202 (0.5411)	0.0482 (0.2185)	0.0546 (0.2185)
	Disease state	0.4595 (0.6127)	0.1415 (0.3774)	0.9219 (0.9361)	0.9361 (0.9361)
Nasal ^¥^	Sampling time point	0.8345 (0.8344)	0.6316 (0.8344)	0.7071 (0.8344)	0.7671 (0.8344)

* All *p*-values were set to significance level 0.01. ^¥^ Nasal samples were only tested against sampling time point due to low incidence of BRD.

## Data Availability

The 16S rRNA gene sequencing data are publicly available in the EBI repository under study accession PRJEB76862 and on the QIITA study platform under study ID 13813 [34].

## Supplementary Materials

The following supporting information can be downloaded at: https://www.mdpi.com/article/10.3390/ani15030361/s1, Table S1: The results from cNST models to test stochasticity and neutrality in early-life microbiomes; Table S2: The total number of calves associated with fecal severity scores at each sampling time point; Figure S1: Mock community assessment; Figure S2. Linear regression models for breed, alpha diversity, and fecal severity; Figure S3. Linear regression models for breed, alpha diversity, and nasal samples; Figure S4. Linear regression models for sex, alpha diversity, and nasal samples; Figure S5. Linear regression models for sampling time points, alpha diversity metrics, and fecal samples; Figure S6. Multiple regression models for fecal and nasal sample types, with predictors of sampling time point, individual calf, and calf age in days for effects on alpha diversity metrics; Figure S7. Linear regression models for fecal severity scores and alpha diversity metrics.

## Abbreviations

The following abbreviations are used in this manuscript:

AKP	Anna Karenina Principles
ANCOM-BC	Analysis of composition of microbiomes with bias corrections
BRD	Bovine respiratory disease
DEMENT	Decomposition Model of Enzymatic Traits
DMM	Dirichlet multinomial model
FDR	False discovery rate
GI	Gastrointestinal
NMDS	Nonmetric multidimensional
NST	Normalized stochasticity testing
NSTi	Normalized stochasticity index
MST	Modified stochasticity ratio
PERMANOVA	Permutational multivariant analysis of variance
β_RC_	Raup–Crick distance
SES	Standardized effect size index
URT	Upper respiratory tract
