# Supplementary material for: Stochasticity Highlights the Development of Both the Gastrointestinal and Upper-Respiratory-Tract Microbiomes of Neonatal Dairy Calves in Early Life
Source: Animals (Basel). 2025 Jan 27;15(3):361. doi: 10.3390/ani15030361 (PMC11816138; doi:10.3390/ani15030361)
Supplement: Supplementary file 1 [file animals-15-00361-s001.zip › animals-3410519-supplementary.pdf]

Table S1. The results from cNST models to test stochasticity and neutrality in early-life microbiomes

Sample Type	NSTi[¥]	MST*	SES[!]	β_{RC}[ø]
Fecal	0.9765	0.5491	0.0576	0.0146
Nasal	0.9603	0.3661	0.0463	0.0166

[¥] NSTi: normalized stochasticity testing index; values greater than 0.5 favor stochasticity

*MST: modified stochasticity ratio; values greater than 50% favor stochasticity

[!] SES: standardized effect size; values > 2 and < -2 indicate determinism; values close to zero indicate neutrality

[ø] Raup-Crick Distance: values approaching 0 favor neutrality

Table S2. The total number of calves associated with fecal severity scores at each sampling time point.

Fecal Severity Score*	Time Point 1	Time Point 2	Time Point 3
Healthy (0)	6	4	7
Mild (1)	10	6	7
Moderate (2)	2	5	3
Severe (3)	1	4	2

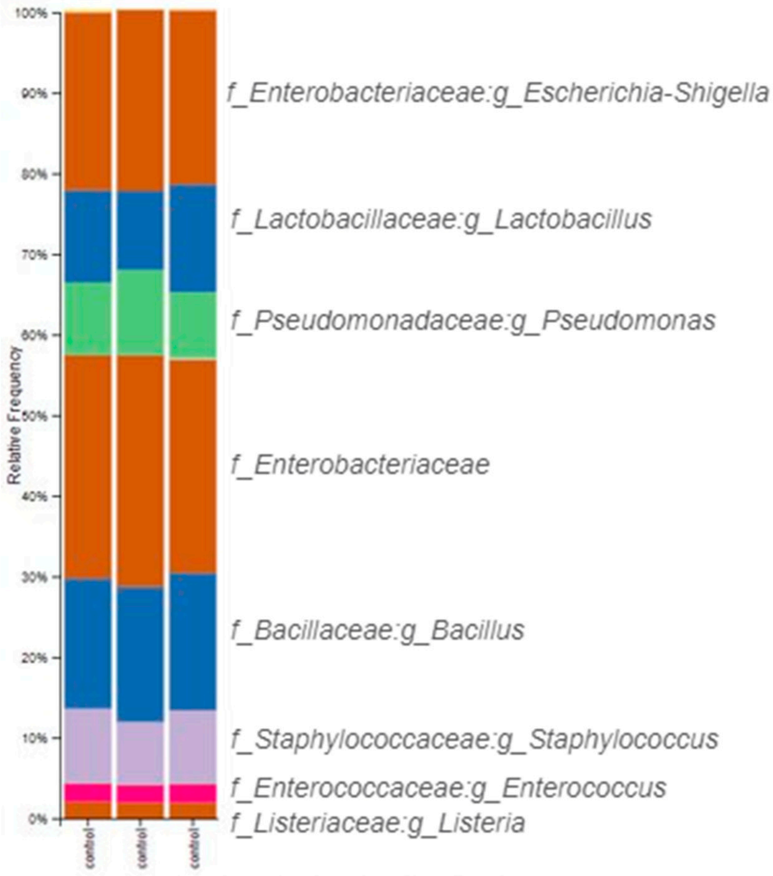

Figure S1. Mock community assessment. The mock communities used as positive communities were assessed for quality control of the dataset. The negative communities (not shown) indicated low sequencing numbers. The positive controls were validated by the mock community make-up and allowed for validation of the dataset and the subsequent removal of the controls for further data processing.

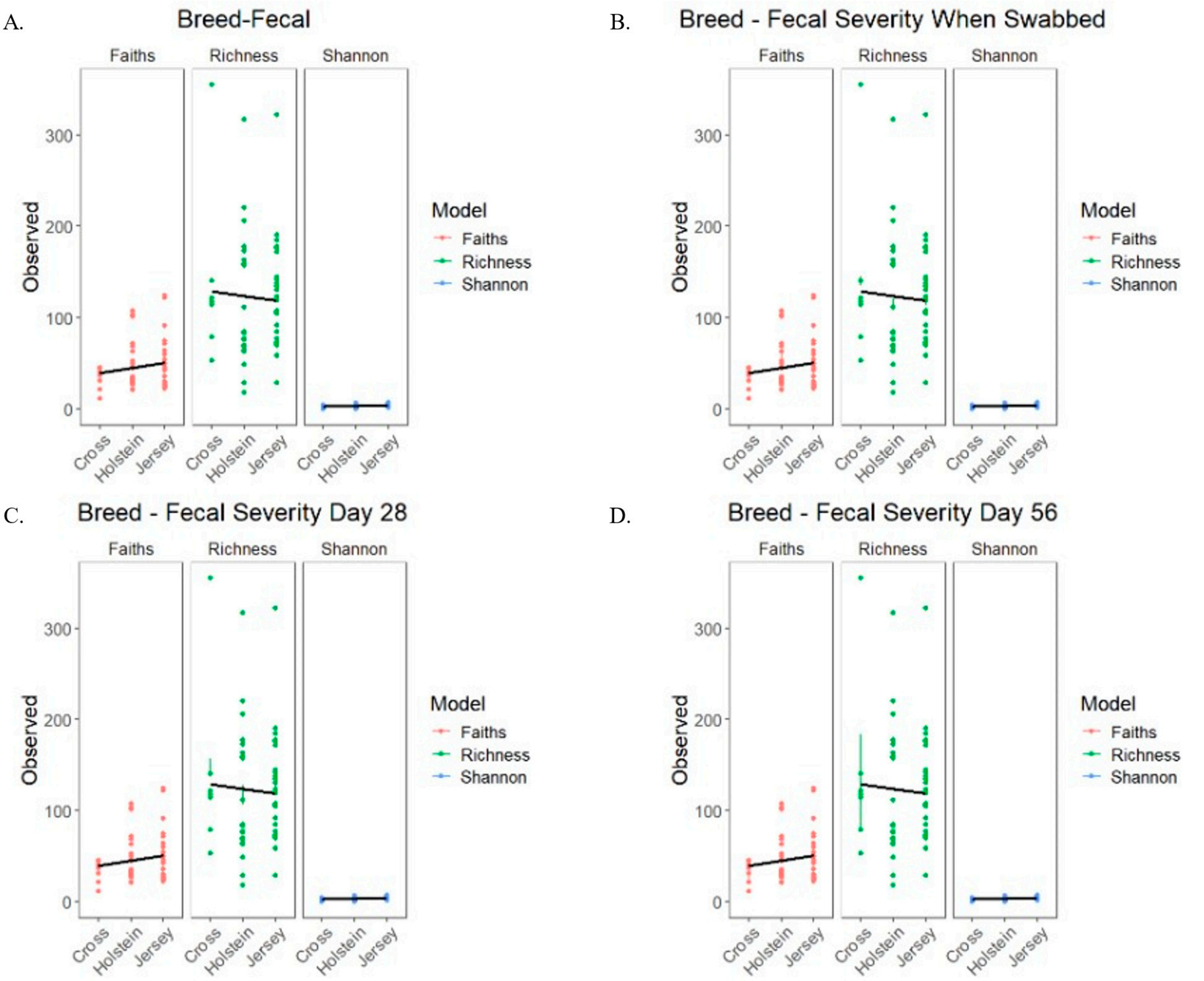

Figure S2. Linear regression models for breed, alpha diversity, and fecal severity. Models were fit to assess the correlation between diversity metrics and animal breed for fecal samples where the observed values for diversity are on the Y axes and the predicted fitted values are on the X axes. A.) Faith's PD (F-statistic = 1.129, p-value = 0.3312, R^2 = 0.04), Shannon's Diversity (F-statistic = 2.664, p-value = 0.079, R^2 = 0.09), and richness (F-statistic = 0.3018, p-value = 0.7407, R^2 = 0.01) were not correlated with breed. In plots B., C., and D., animal breed and fecal severity scores were assessed for correlations with diversity metrics. B.) Faith's PD (F-statistic = 0.9081, p-value = 0.4835, R^2 = 0.08), Shannon's Diversity (F-statistic = 1.4, p-value = 0.2408, R^2 = 0.12) and richness (F-statistic = 0.1582, p-value = 0.9765, R^2 = 0.01) were not correlated with breed and fecal severity at initial swabbing. C.) Faith's PD (F-statistic = 0.7394, p-value = 0.5334, R^2 = 0.04), Shannon's Diversity (F-statistic = 2.163, p-value = 0.1038, R^2 = 0.11). and richness (F-statistic = 0.4888, p-value = 0.6916, R^2 = 0.02) were not correlated with breed and fecal severity at day 28. D.) Faith's PD (F-statistic = 0.9228, p-value = 0.4365, R^2 = 0.05), Shannon's Diversity (F-statistic = 1.749, p-value = 0.1688, R^2 = 0.09), and richness (F-statistic = 0.4888, p-value = 0.6916, R^2 = 0.02) were not correlated with breed and fecal severity at day 56.

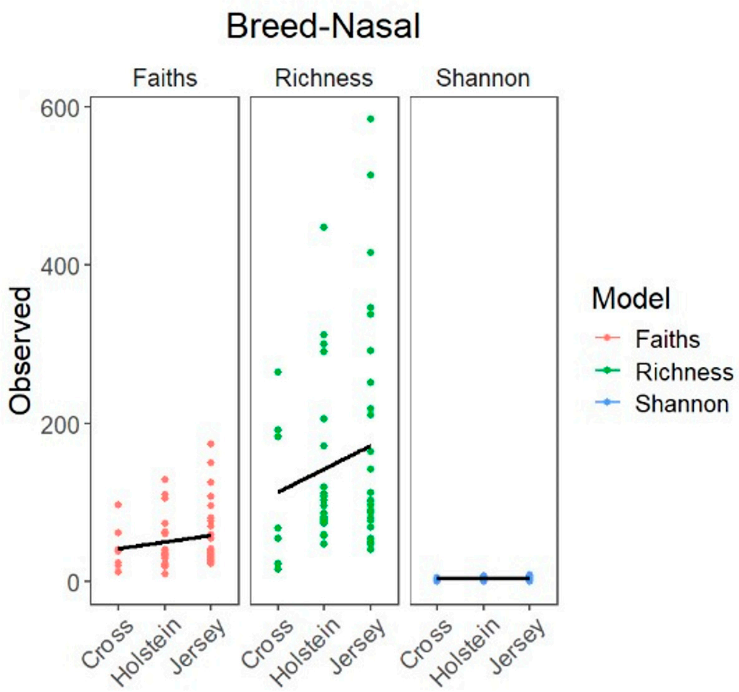

Figure S3. Linear regression models for breed, alpha diversity, and nasal samples. Models were fit to assess the correlation between diversity metrics and animal breed for nasal samples where the observed values for diversity are on the Y axes and the predicted fitted values are on the X axes. Faith's PD (F-statistic = 0.6678, p-value = 0.5172, R^2 = 0.02), Shannon's Diversity (F-statistic = 0.05728, p-value = 0.9444, R^2 = 0.002) and richness (F-statistic = 0.6754, p-value = 0.5134, R^2 = 0.02) were not correlated with breed for nasal samples.

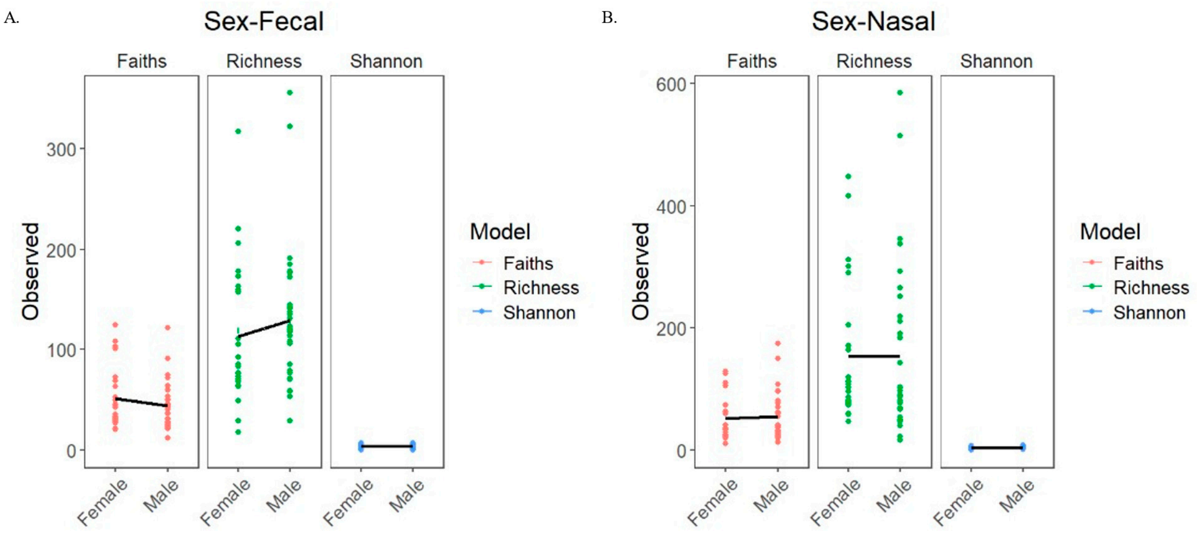

Figure S4. Linear regression models for sex, alpha diversity, and nasal samples. Model were fit to assess the correlation between diversity metrics and animal sex for fecal and nasal samples where the observed values for diversity are on the Y axes and the predicted fitted values are on the X axes. A.) Faith's PD (F-statistic = 1.017, p-value = 0.3179, R^2 = 0.01), Shannon's Diversity (F-statistic = 0.1654, p-value = 0.6859, R^2 = 0.003), and richness (F-statistic = 0.6984, p-value = 0.4071, R^2 = 0.01) for fecal samples were not correlated with sex. B.) Faith's PD (F-statistic = 0.08815, p-value = 0.7677, R^2 = 0.001), Shannon's Diversity (F-statistic = 0.02801, p-value = 0.8677, R^2 = 0.0005), and richness (F-statistic = 0.0001, p-value = 0.9902, R^2 = 2.8e-6) for nasal samples were not correlated with sex.

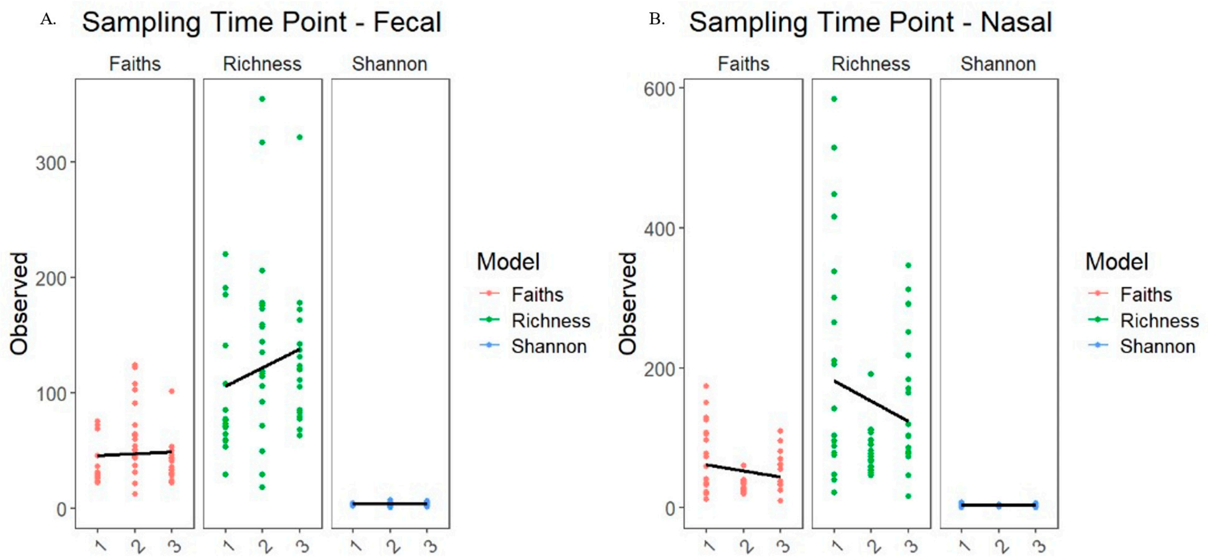

Figure S5. Linear regression models for sampling time points, alpha diversity metrics, and fecal samples. Models were fit to assess the correlation between diversity metrics and sampling time points where the observed values for diversity are on the Y axes and the predicted fitted values are on the X axes. A.) Faith's PD (F-statistic = 6.51, p-value = 0.002, $R^2 = 0.2$) for fecal samples was correlated with sampling time points. Specifically, sampling time point 2 (p-value = 0.0018) was significantly correlated with changes in Faith's PD. Shannon's Diversity (F-statistic = 0.3591, p-value = 0.7, $R^2 = 0.01$) and richness (F-statistic = 2.406, p-value = 0.1001, $R^2 = 0.08$) for fecal were not correlated with sampling time points. B.) Faith's PD (F-statistic = 7.469, p-value = 0.001, $R^2 = 0.22$) and richness (F-statistic = 6.775, p-value = 0.002, $R^2 = 0.2$) for nasal samples were significantly correlated with sampling time points. Specifically, sampling time point 2 was significantly correlated with changes in Faith's PD (p-value = 0.0003) and richness (p-value = 0.0005). Shannon's Diversity (F-statistic = 0.1158, p-value = 0.8909, $R^2 = 0.004$) for nasal samples was not correlated with sampling time points.

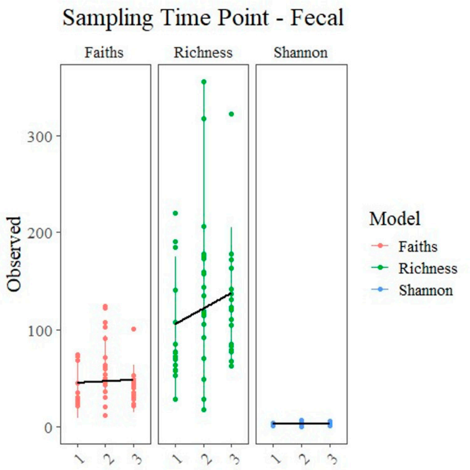

A.

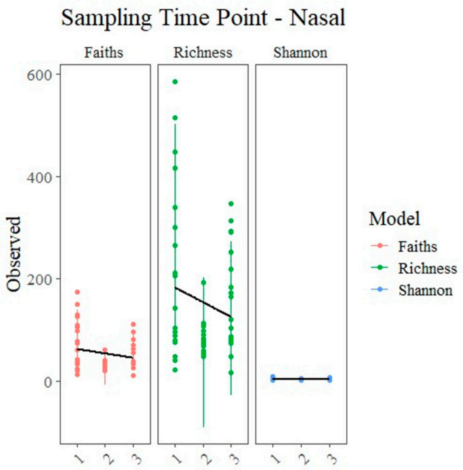

B.

Figure S6. Multiple regression models for fecal and nasal sample types with predictors sampling time point, individual calf, and calf age in days for effects on alpha diversity metrics. Models were fit to assess the correlation between diversity metrics and the predictors sampling time point, individual calf, and calf age in days where the observed values for diversity are on the Y axes and the predicted fitted values are on the X axes. Panel A.) Faith's PD (F-statistic = 1.224, p-value = 0.3033, R^2 = 0.57), Shannon's Diversity (F-statistic = 1.119, p-value = 0.3883, R^2 = 0.55) and richness (F-statistic = 1.428, p-value = 0.182, R^2 = 0.61) were not correlated with the predictors for fecal samples. Panel B.) Faith's PD (F-statistic = 1.325, p-value = 0.2369, R^2 = 0.59) and Shannon's Diversity (F-statistic = 0.5291, p-value = 0.949, R^2 = 0.36) were not correlated with the predictors for nasal samples. However, richness (F-statistic = 2.343, p-value = 0.01, R^2 = 0.72) was correlated with the predictors for nasal samples.

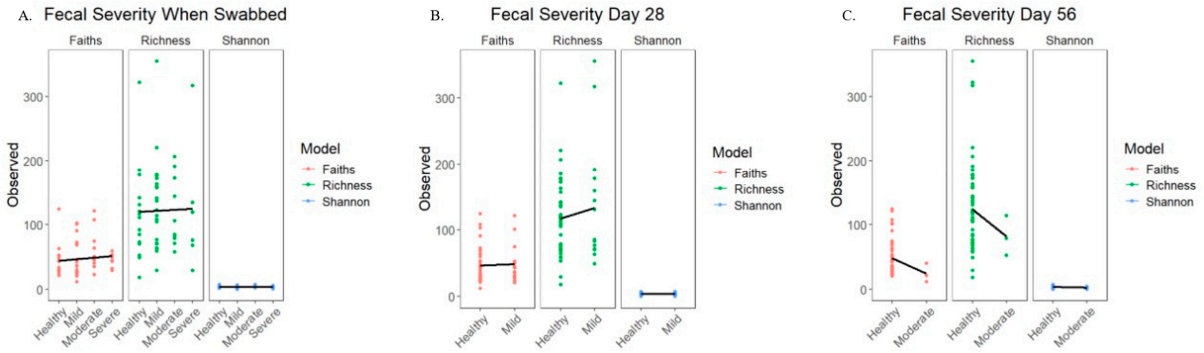

Figure S7. Linear regression models for fecal severity scores and alpha diversity metrics. Models were fit to assess the correlation between diversity metrics and fecal severity scores where the observed values for diversity are on the Y axes and the predicted fitted values are on the X axes. A.) Faith's PD (F-statistic = 0.804, p-value = 0.4974, R^2 = 0.04), Shannon's Diversity (F-statistic = 0.614, p-value = 0.609, R^2 = 0.03) and richness (F-statistic = 0.05296, p-value = 0.9837, R^2 = 0.003) were not correlated with fecal severity scores at initial sampling. B.) Faith's PD (F-statistic = 0.05933, p-value = 0.8085, R^2 = 0.001), Shannon's Diversity (F-statistic = 0.9505, p-value = 0.334, R^2 = 0.01) and richness (F-statistic = 0.5822, p-value = 0.4488, R^2 = 0.01) were not correlated with fecal severity at day 28. C.) Faith's PD (F-statistic = 2.338, p-value = 0.1322, R^2 = 0.04), Shannon's Diversity (F-statistic = 1.325, p-value = 0.2549, R^2 = 0.02) and richness (F-statistic = 1.025, p-value = 0.3159, R^2 = 0.01) were not correlated with fecal severity at day 56.
